# AmCBF1 Transcription Factor Regulates Plant Architecture by Repressing *GhPP2C1* or *GhPP2C2* in *Gossypium hirsutum*

**DOI:** 10.3389/fpls.2022.914206

**Published:** 2022-05-30

**Authors:** Junchao Lu, Lihua Wang, Qianqian Zhang, Caixia Ma, Xiaofeng Su, Hongmei Cheng, Huiming Guo

**Affiliations:** ^1^Zhengzhou Research Base, State Key Laboratory of Cotton Biology, School of Agricultural Sciences, Zhengzhou University, Zhengzhou, China; ^2^Biotechnology Research Institute, Chinese Academy of Agricultural Sciences, Beijing, China; ^3^College of Agriculture, Shanxi Agricultural University, Taigu, China

**Keywords:** cotton, transcription factor, *AmCBF1*, *GhPP2C*, plant architecture and plant height

## Abstract

Dwarfism is a beneficial trait in many crops. Dwarf crops hold certain advantages over taller crops in lodging resistance, fertilizer tolerance, and yield. Overexpression of CBF/DREB transcription factors can lead to dwarfing in many plant species, but the molecular mechanism of plant dwarfing caused by overexpression of CBF/DREB in upland cotton (*Gossypium hirsutum*) remains unclear. In this study, we observed that overexpression of the *Ammopiptanthus mongolicus* AmCBF1 transcription factor in upland cotton R15 reduced plant height, whereas virus-induced gene silencing of *AmCBF1* in the derived dwarf lines L28 and L30 partially restored plant height. Five protein phosphatase (PP2C) genes (*GhPP2C1* to *GhPP2C5*) in cotton were identified by RNA-sequencing among genes differentially expressed in L28 or L30 in comparison with R15 and thus may play an important role in AmCBF1-regulated dwarfing in cotton. Gene expression analysis showed that the *GhPP2C* genes were down-regulated significantly in L28 and L30, and silencing of *GhPP2C1* or *GhPP2C2* in R15 inhibited the growth of cotton seedlings. Subcellular localization assays revealed that GhPP2C1 was localized to the cell membrane and nucleus, whereas GhPP2C2 was exclusively localized to the nucleus. Yeast one-hybrid and dual-luciferase assays showed that AmCBF1 was able to bind to the CRT/DRE elements of the upstream promoter of *GhPP2C1* or *GhPP2C2* and repress their expression. These findings provide insight into the mechanism of dwarfing and may contribute to the breeding of dwarf cultivars of upland cotton.

## Introduction

Dwarfism is a valuable agronomic trait, as it helps to improve the light use efficiency of the population and increases the ability of plants to resist an adverse external environment (Khush, 1999). In agricultural and horticultural crops, breeding for dwarfism is particularly important. Some tall crops have weak stalks and are susceptible to lodging, whereas dwarf crops have strong stalks and are resistant to lodging, highly fertilizer tolerant, and produce significantly increased yields through reasonably dense planting. Dwarf and compact horticultural plants are increasingly popular with consumers because of their high ornamental value. In recent years, considerable progress has been made in screening and identifying genes associated with plant dwarfing. Phytohormone-related genes play an important role in causing dwarfing in plants (Hu et al., 2017; Li et al., 2018; Graeff et al., 2020). In addition, genes that affect plant cell wall synthesis, such as a mutation of the cellulose biosynthesis gene *CESA*, or regulation by transcription factors such as C-repeat binding factor/dehydration responsive element-binding factor (CBF/DREB) proteins, can cause dwarfing in plants (Xie et al., 2011). In cotton, the dwarf variety AS98 was detected in an introgressed line derived from an interspecific hybrid and exogenous application of gibberellic acid (GA_3_) restores the plant height phenotype (Zhang et al., 2011; Jia et al., 2016). In the cotton *pag1* mutant, cell growth is disrupted, resulting in a dwarf phenotype and reduced fiber quality (Yang et al., 2014). The contents of indoleacetic acid and abscisic acid (ABA) are reduced in the Asian cotton dwarf *sd^a^* mutant, and genetic analysis indicates that the dwarfism trait is controlled by a pair of recessive genes (Wu et al., 2009). The aforementioned studies indicate that utilization of a variety of dwarfing resources provides a means to increase cotton yield by changing the plant architecture.

The CBF proteins comprise a subfamily of the APETALA 2/ETHYLENE-RESPONSIVE FACTOR (AP2/ERF) family of transcription factors, also termed DREBs, which are involved in a range of processes during the plant life cycle (Agarwal et al., 2006). The CBF protein binds to the C-repeat/dehydration-responsive element (CRT/DRE) sequence in the promoter of downstream genes to regulate their expression, and enhances plant resistance to a variety of stresses, such as drought, freezing, and salinity (Baker et al., 1994; Yamaguchi-Shinozaki and Shinozaki, 1994; Dong et al., 2006). Overexpression of CBF transcription factors in diverse plants affects plant growth and leads to dwarfing, while affecting plant stress resistance. For example, overexpression of *AtDREB1A* or *OsDREB1A/2A* in *Arabidopsis* improves cold and drought tolerance, but growth of transgenic *Arabidopsis* is severely inhibited (Liu et al., 1998; Dubouzet et al., 2003). Similarly, overexpression of *OsDREB1A/1B* in rice (*Oryza sativa*) results in dwarfing (Ito et al., 2006). Interestingly, application of exogenous gibberellin (GA) partially restores the height of plants overexpressing *CBF*, suggesting that dwarfing caused by *CBF* overexpression may be due to GA deficiency (Hsieh et al., 2002). Overexpression of *DgDREB1B* in *Arabidopsis* leads to reduction in plant height and significant up-regulation of *GA2ox7* and GA-inactivating enzymes, suggesting that *DgDREB1B* may be involved in GA-mediated development in plants (Tong et al., 2009). Twenty-one *CBF* genes have been identified in the upland cotton (*Gossypium hirsutum*) genome and are categorized into four groups: GhCBF I, GhCBF II, GhCBF III, and GhCBF IV (Ma et al., 2014). Overexpression of *GhDREB1* in tobacco (*Nicotiana tabacum*) causes retarded growth and delays flowering (Shan et al., 2007). Recently, it was shown that overexpression of *GhDREB1B* in upland cotton results in significant reduction in plant height, shorter branch lengths, and reduced branch angles (Ji et al., 2021). These results confirm that overexpression of CBF/DREB transcription factors can lead to plant dwarfing, but the specific mechanism of dwarfing remains to be studied.

Post-translational modification of proteins plays an important role in plant developmental processes. Among these modifications, protein phosphorylation/dephosphorylation plays a role at various stages of plant growth and development by regulating protein activity and participating in the transmission of a number of signals in plants (Schweighofer et al., 2007; Fuchs et al., 2013; Singh et al., 2016). This reversible protein phosphorylation is achieved through the activities of the enzyme pair protein kinase and protein phosphatase. Depending on the catalytic substrate, protein phosphatases are mainly divided into protein tyrosine phosphatases and protein serine/threonine phosphatases (Xin et al., 2021). Protein phosphatase 2C (PP2C) comprises a class of protein serine/threonine phosphatases and is a monomeric enzyme (Zhang et al., 2022). In plants, PP2C is less conserved at the N terminus and more conserved at the C terminus, which confers PP2C with functional diversity (Ingebritsen and Cohen, 1983; Hubbard and Cohen, 1993; Bork et al., 1996). A recognized mechanism of action is that PP2C participates in the ABA signaling pathway. In this signaling mechanism, PP2Cs bind to SnRK2, resulting in the loss of SnRK2 phosphokinase activity and the inability of downstream substrates to be phosphorylated (Soon et al., 2012). In the presence of ABA, the ABA receptor PYR/PYL/RCAR binds to PP2C and SnRK2 is released, phosphorylating downstream transcription factors, such as ABA-responsive element-binding factors, and further activating the expression of certain downstream genes (Miyazono et al., 2009; Cutler et al., 2010; Hubbard et al., 2010; Rodrigues et al., 2013; Zhu, 2016).

The function of *PP2C* genes is closely associated with processes such as signal transduction, growth and development, hormone regulation, and oxidative stress (Gaits et al., 1997; Rodriguez, 1998; Kerk et al., 2002; Schweighofer et al., 2004). Nine and 10 subgroup A PP2C members have been identified in *Arabidopsis* and rice, respectively, of which most play a negative regulatory role in ABA signaling (Saez et al., 2004; Kuhn et al., 2006; Nishimura et al., 2007; Bhaskara et al., 2012; He et al., 2014; Née et al., 2017; Miao et al., 2018). Overexpression of *OsPP108* in *Arabidopsis* enhances plant tolerance to salt and drought stress, and reduces ABA sensitivity (Singh et al., 2015). Overexpression of *OsPP2C09* in rice results in rapid growth and increases individual plant yield, whereas its mutant reduces plant height and yield (Miao et al., 2020). A lack of *SlPP2C3* expression in tomato (*Solanum lycopersicum*) not only affects plant growth, but also accelerates the onset of fruit ripening and affects fruit gloss by altering the outer epidermal structure (Liang et al., 2021). Genome-wide identification of PP2C family members detected 87, 99, 147, and 181 *PP2C* genes in *Gossypium arboreum*, *Gossypium raimondii*, *Gossypium barbadense*, and *G. hirsutum*, respectively. Among these genes, the 181 PP2Cs in *G. hirsutum* were roughly divided into 12 subgroups; the transcription of some of these *PP2C* genes was induced by high temperature, low temperature, drought, or high salt stress separately, suggesting that *PP2C* genes play a crucial role in abiotic stress response in cotton (Shazadee et al., 2019). However, the relationship between *PP2C* genes and a dwarf phenotype in upland cotton has not been examined.

Cotton, which is among the world’s most important oilseed and textile crops, plays an important role in global agricultural and industrial production. With technological developments, mechanized harvesting of cotton is increasingly important and dwarf cotton is a major breeding target on account of the compact plant height and suitability for machine harvesting. Although dwarf cotton varieties have various advantages, there are still relatively few dwarf cotton varieties on the market today. Therefore, development of new cotton varieties showing stable inheritance of dwarfism is a matter of urgency. Although a number of dwarfing-related genes have been identified, little information is available on their biological functions, especially the molecular mechanisms that regulate cotton growth and development.

In our previous work, *Ammopiptanthus mongolicus AmCBF1* was overexpressed in the upland cotton line R15 to evaluate its function in abiotic stress response. We observed that overexpression of *AmCBF1* increased the resistance of cotton to low temperature and drought stress, and decreased the plant height. In the present study, we further confirmed the relationship between *AmCBF1* and the dwarf phenotype in cotton. Five down-regulated *GhPP2C* (*GhPP2C1-GhPP2C5*) genes were identified among differentially expressed genes by RNA-Seq and AmCBF1 transcription factors could negatively regulate the expression of *GhPP2C1* or *GhPP2C2*. These results will help to understand the molecular mechanism of cotton dwarf and provide theoretical basis for cotton dwarf breeding.

## Materials and Methods

### Plant Materials and Growing Conditions

Transgenic dwarf cotton lines (L28 and L30), transgene recipient (R15), and tobacco (*Nicotiana benthamiana*) plants were grown in pots containing nutrient soil in a greenhouse (25 ± 3°C, 50% relative humidity, and 16 h light/8 h dark cycle). In addition, R15, L28, and L30 were grown in the field (39°36′10.3”N，116°36′07.3″E). Cotton and tobacco plants at different growth stages were selected for subsequent experiments.

### Quantitative Real-Time PCR

Total RNA was extracted from cotton leaves using an RNA extraction kit (Tiangen Biotech, Beijing, China). The RNA concentration was measured using a NanoDrop 2000 spectrophotometer (Thermo Fisher Scientific, Waltham, MA, United States). Purified RNA (>1 μg) treated with gDNase was reverse transcribed with HiScript III RT SuperMix for qPCR (+gDNA wiper; Vazyme Biotech, Nanjing, China) to generate the first-strand cDNA. Gene expression levels were analyzed with an ABI ViiA™ 7 Real-Time PCR System (Applied Biosystems, Waltham, MA, United States) and ChamQ Universal SYBR qPCR Master Mix (Vazyme Biotech). The quantitative real-time PCR (qRT-PCR) protocol was as follows: 40 cycles at 95°C for 30 s, 95°C for 10 s, 60°C for 30 s, and 72°C for 15 s. Relative expression levels were calculated using the 2^−ΔΔ*C*t^ method. Primers were designed using PRIMER PREMIER 5 (Premier Biosoft, Palo Alto, CA, United States). The cotton *ubq* gene (Gh_D13G1489) was used as an internal control.

### Virus-Induced Gene Silencing

Virus-induced gene silencing (VIGS) is an approach widely used to study gene function and functional genomics in a variety of plants (Becker and Lange, 2010). We performed a VIGS experiment to test if plant height was partly restored after silencing *AmCBF1* in L28 or L30. The *AmCBF1* fragment was amplified and inserted into the pTRV2 vector digested with the *Xba*I and *Kpn*I restriction enzymes. The plasmids pTRV1, pTRV2, pTRV2-*AmCBF1*, and pTRV2-*CLA1* were transferred to *Agrobacterium tumefaciens* strain GV3101 receptor cells by electroshock and incubated overnight in LB liquid medium supplemented with 50 mg L^−1^ kanamycin, 50 mg L^−1^ rifampicin, 10 mM MES, and 20 mM acetosyringone at 28°C on a shaker at 200 rpm. The *Agrobacterium* cells were collected by centrifugation and suspended in permeate [containing 10 mM MgCl_2_, 10 mM MES (pH 5.6), and 20 μM acetyleugenone] to optical density (OD_600_) = 1.2. After incubation at room temperature for 3 h in the dark, the suspensions containing the pTRV2, pTRV2-*AmCBF1*, or pTRV2-*CLA1* plasmids were mixed with pTRV1 suspension (1:1, *v*/*v*) respectively. Cotton seedlings with well-grown, fully expanded cotyledons were selected and a small wound was made on the lower surface of the cotyledons with a syringe needle. Silencing of the *CLA1* gene in R15 was used as a positive control to produce an albino phenotype in cotton. The injected cotton plants were cultured in the dark for 24 h, then for 4 weeks under a 16 h light/8 h dark cycle at 23°C and 50% relative humidity. Total RNA was extracted from young cotton leaves and qRT-PCR was performed to detect the relative expression level of *AmCBF1*. In addition, based on sequence alignment of *GhPP2C1*, *GhPP2C2*, and *GhPP2C3*, we designed specific VIGS fragments and qRT-PCR detection fragments (Supplementary Figure 3). The *GhPP2C1*, *GhPP2C2*, or *GhPP2C3* genes were silenced in R15 by VIGS to investigate whether the genes affect the height of cotton plants. Fragments for silencing *GhPP2C1*, *GhPP2C2*, and *GhPP2C3* were amplified from R15 cotton cDNA using specific primers and inserted into the *Xba*I and *Kpn*I double-cleaved pTRV2 vector using seamless cloning. The specific experimental method was based on the gene silencing analysis of *AmCBF1*. The primers used for VIGS vector construction and qRT-PCR detection are listed in Supplementary Table 1.

### Scanning Electron Microscopy

To investigate whether overexpression of *AmCBF1* affects the morphology and size of cotton leaf cells, the upper epidermis of cotton seedling leaves was observed by scanning electron microscopy. The fourth true leaf of cotton seedlings was washed three times with deionized water and fixed with 2.5% glutaraldehyde at 4°C for more than 8 h. After dehydration, the fixed samples were dried in a CO_2_ critical-point desiccator for approximately 2 h. The samples were observed under a scanning electron microscope (Hitachi SU-8010, Tokyo, Japan) and photographed.

### RNA-Sequencing Analysis

Total RNA extracted from fresh seedlings of R15, L28, and L30 was subjected to transcriptome sequencing by Libaijia Biologicals (Beijing, China). Two biological replicates of each material were designated R15R1, R15R2, L28R1, L28R2, L30R1, and L30R2. Quantification of gene expression levels was expressed as fragments per kilobase of transcript per million fragments mapped (FPKM). The fold change is the ratio of expression between the two samples, and the false discovery rate (FDR) is a value obtained by correcting for the difference in significance *p*-value (*p*-value). Fold change ≥2 and FDR < 0.01 were used as the screening criteria. The Benjamini–Hochberg correction method was used to correct the *p*-value obtained from the original hypothesis test to avoid the problem of false positives. Finally, the FDR was adopted as the crucial index for screening differentially expressed genes (DEGs). Twelve DEGs involved in different metabolic pathways were randomly selected to verify the transcriptome data. The selected genes, designated Q1–Q12, are listed in Supplementary Table 2.

### Phylogenetic Analysis

The amino acid sequence of all PP2C proteins of upland cotton were downloaded from the Cotton Functional Genomics Database (cottonFGD).^1^ Sequences of the PP2C proteins of *Arabidopsis* were downloaded from The Arabidopsis Information Resource database (TAIR).^2^ Phylogenetic analyses were conducted using the neighbor-joining method implemented in the MEGA 7.0 software.

### Subcellular Localization

The full-length coding regions (without the termination codon) of *GhPP2C1*, *GhPP2C2*, and *GhPP2C3* were inserted into the pCambia1302 vector carrying the green fluorescent protein (GFP) gene. The plasmids 1302, 1302-GhPP2C1, 1302-GhPP2C2, and 1302-GhPP2C3 were transferred into *A. tumefaciens* strain EHA105 using the freeze–thaw method, and incubated in LB liquid medium supplemented with 50 mg L^−1^ kanamycin and 50 mg L^−1^ rifampicin for 16 h at 28°C on a shaker at 200 rpm. The *Agrobacterium* cells were centrifuged at 3,000 × *g* using a High-speed Micro Centrifuge and the supernatant was discarded. The sediment was resuspended in MMA solution [containing 10 mM MgCl_2_, 10 mM MES (pH 5.6), and 150 μM acetyleugenone] to OD_600_ = 1.2 and incubated at room temperature for 3 h in the dark. Healthy tobacco leaves were selected and the suspension was gently injected into the lower surface of the leaves with a syringe. Injection of GFP alone was used as a control, and H2B-mCherry was used as a nucleolus marker. After incubation for 16 h in the dark at 21°C, the leaves were incubated in the light for 8 h. Two days after injection, the fluorescence signal was observed using a LSM 700 confocal microscope (Zeiss, Oberkochen, Germany). The primers used for construction of the transient expression vectors are listed in Supplementary Table 1.

### Yeast One-Hybrid assay

Three or two predicted CRT/DRE (A/GCCC/GAC) elements were identified in the 3 kb promoter regions upstream of the *GhPP2C1* or *GhPP2C2* genes, and designated P1, P2, P3, P4, and P5, respectively. A Y1H analysis was conducted to verify whether AmCBF1 is capable of interacting with the corresponding *cis*-elements in the upstream promoters of *GhPP2C1* and *GhPP2C2*. The full-length coding sequence of *AmCBF1* was amplified using specific primers and inserted into the pGADT7 vector digested with *Eco*RI and *Bam*HI to generate the prey plasmid. The 2,049 bp or 882 bp promoter region containing all CRT/DRE elements of *GhPP2C1* or *GhPP2C2* was separately cloned and inserted into the pAbAi vector digested with *Kpn*I and *Sal*I to generate the bait plasmid. In addition, separate forward and reverse complementary oligonucleotide strands were synthesized for P1, P2, P3, P4, and P5, annealed to generate promoter DNA fragments containing the CRT/DRE elements, and inserted into the pAbAi bait vector. The bait plasmid was transformed into the yeast strain Y1H Gold by *Bstb*I digestion using the EX-Yeast Transformation Kit (Zhuangmeng Bio, Beijing, China). The linearized bait plasmid was homologously integrated with the yeast Y1H Gold genome to generate a decoy reporter yeast strain, which was tested using an aureobasidin A (AbA) inhibition assay and Matchmaker Insert Check PCR Mix 1 (Clontech Laboratories, Inc., Palo Alto, CA, United States) to identify positive strains. The prey plasmids were then transformed into the bait reporter strains to verify DNA–protein interactions. The transformed cells were cultured on SD/−Leu/−Ura medium incubated for 3 days at 30°C. Positive strains were identified using the pGADT7 universal primer. Subsequently, yeast cells at different dilutions (1:10, 1:100, and 1:1,000) were cultured on SD/−Leu/−Ura medium supplemented with different concentration of AbA (0, 200, or 400 ng ml^−1^). The pGADT7 vector was used as a negative control. The primers used in this experiment are listed in Supplementary Table 1.

### Dual-Luciferase Analysis

The full-length coding sequence of *AmCBF1* was inserted by seamless cloning into the *Nco*I monozyme-cleaved pCambia1302 vector to generate the effector plasmids. A short truncated fragment of the promoter containing different numbers of CRT/DRE elements upstream of the ATG start codon for *GhPP2C1* or *GhPP2C2* was inserted by seamless cloning into the pGreenII 0800-LUC vector to generate GhPP2C1 Pro:LUC, C1:LUC, C2:LUC, GhPP2C2 Pro:LUC, and C3:LUC reporter plasmids. The recombinant plasmid and the negative control plasmid were introduced into *Agrobacterium* EHA105 (pSoup-p19) competent cells using the freeze–thaw method, inoculated in LB liquid medium supplemented with 50 mg L^−1^ kanamycin and 50 mg L^−1^ rifampicin, and incubated at 28°C on a shaker at 200 rpm to OD_600_ = 1.0. The *Agrobacterium* cells were centrifuged at 3,000 × *g* for 10 min. The supernatant was discarded and the *Agrobacterium* cells were suspended in permeate [containing 10 mM MgCl_2_, 10 mM MES (pH 5.6), and 150 μM acetosyringone] to OD_600_ = 1.2 and incubated for 3 h at room temperature in the dark. The suspensions were mixed (1:1, *v*/*v*) and injected into the lower surface of the tobacco leaves using a needleless syringe and incubated for 48–60 h under a 16 h light/8 h dark cycle at 23°C and 50% relative humidity. D-Luciferin potassium salt (10 μM) was sprayed onto the tobacco leaves and then imaged using the LB985 Night SHADE fluorescence imaging system (Berthold Technologies, Bad Wildbad, Germany). The dual LUC activity of the different samples was determined using a GloMax 20/20 luminometer (Promega, Madison, WI, United States) and the Dual-Luciferase Reporter Assay System (Promega) in accordance with the manufacturer’s instructions. The primers used in this experiment are listed in Supplementary Table 1.

### Statistical Analysis

Statistical analyses were performed with the SPSS 22.0 software (IBM, Armonk, NY, United States). Values were the means ± SE deviations. Comparisons between groups were performed using one-way ANOVA followed by Duncan’s multiple comparisons. For all comparisons, significant at *p* < 0.05 and highly significant at *p* < 0.01 were considered statistically significant.

## Results

### *AmCBF1* Is Closely Associated With Plant Height

**Figure 1:**
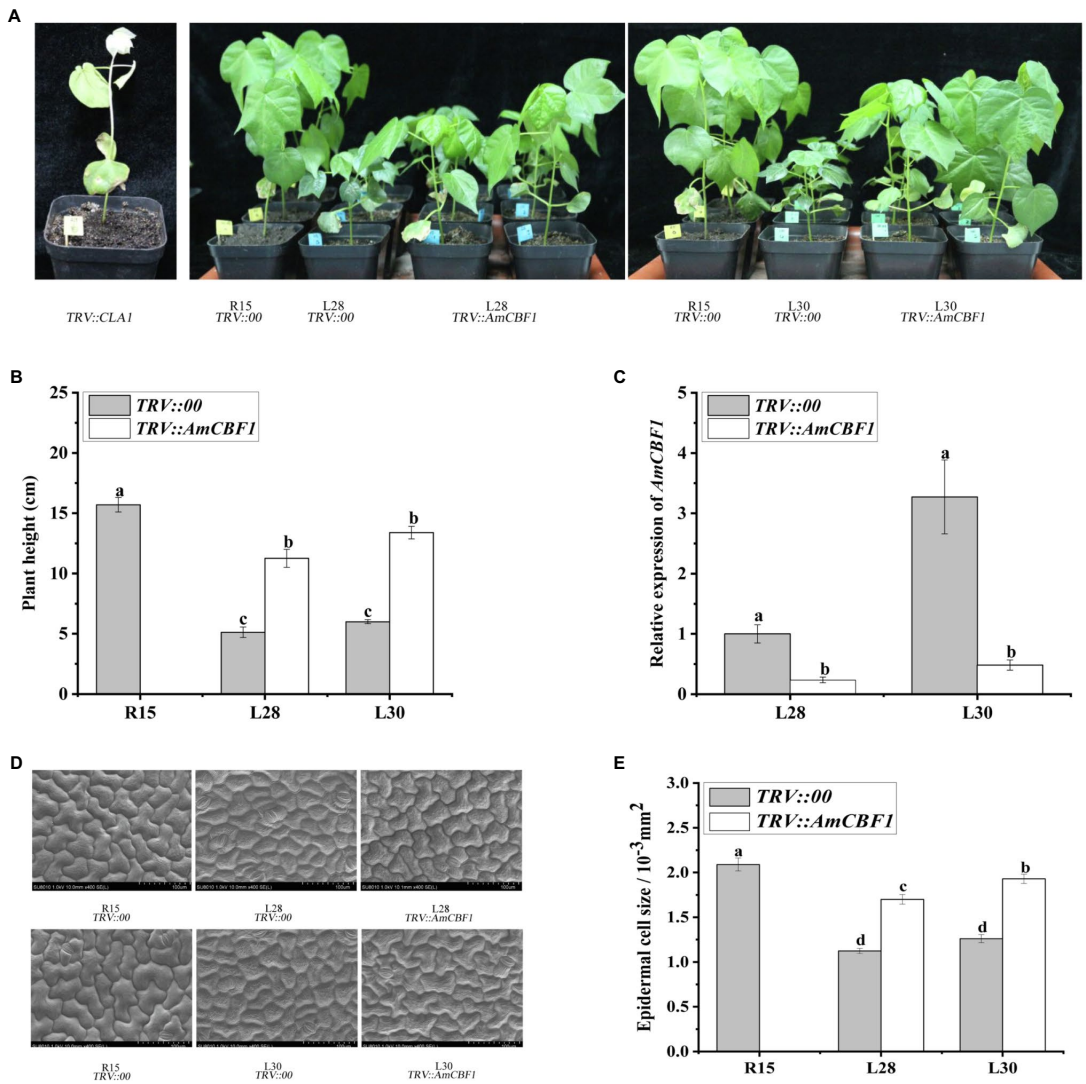
Cotton phenotypes and leaf epithelial cells observed before and after silencing of *AmCBF1* by VIGS. **(A)** Phenotype of cotton after *AmCBF1* silencing. TRV::*CLA1*, albino phenotype, positive control; TRV::*AmCBF1*, *AmCBF1* silencing in L28 and L30. **(B)** Cotton plant height after *AmCBF1* silencing. **(C)** Relative expression of *AmCBF1* in cotton leaves after *AmCBF1* silencing. **(D,E)** Scanning electron micrographs and size of the upper epidermal cells of true leaves. Error bars indicate the standard error (*n* = 3 biological replicates). Different lower-case letters above bars indicate a significant difference at *p* < 0.05 as determined using Duncan’s multiple range test.

The plant height of upland cotton overexpressing *AmCBF1* was significantly reduced in both the greenhouse and the field. During the growth period, the plant height of L28 and L30 decreased by approximately 33 and 35% compared with that of R15 (Supplementary Figure 2). In the VIGS experiment, silencing of the cotton *CLA1* gene in R15 was used as a positive control (Figure 1A). Silencing of *AmCBF1* in L28 or L30 reduced the expression of *AmCBF1* by approximately 75 and 85%, and the plant height was partly restored to 70.5 and 81.3% of that of R15, respectively (Figures 1A–C). The plant height of VIGS lines may be depending on the silencing efficiency of *AmCBF1*. These results indicated that overexpression of *AmCBF1* was crucial for dwarfing in the cotton lines.

### *AmCBF1* Negatively Regulates the Size of Cotton Leaf Epidermal Cells

The epidermal cells were observed with a scanning electron microscope. The epidermal cells of true leaves of L28 and L30 were smaller than those of R15, and the cells were more tightly packed. After silencing of *AmCBF1* in L28 and L30, the epidermal cells of true leaves tended to enlarge, and partially recovered the size and morphology of the epidermal cells of R15 (Figure 1D). Statistical analysis indicated that the size of epidermal cells in L28 and L30 decreased by approximately 40 and 45% compared with that of R15, and the size of epidermal cells was recovered by approximately 25 and 35%, respectively, after silencing of *AmCBF1* (Figure 1E). Overexpression of *AmCBF1* led to reduction in the size of leaf epidermal cells, which suggested that the dwarfing mechanism in cotton may be regulated by AmCBF1.

### RNA-Seq Analysis

**Figure 2:**
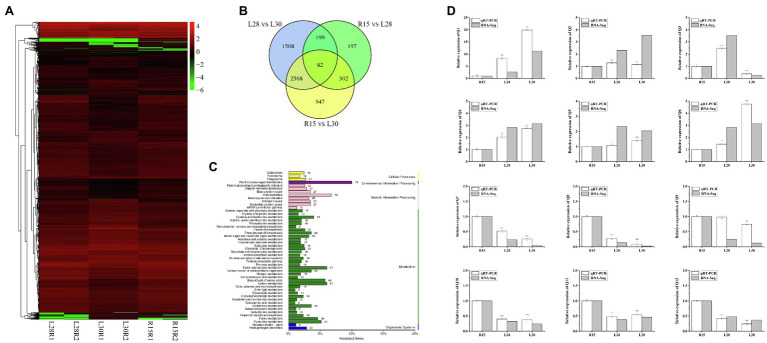
RNA-seq analysis of cotton R15, L28, and L30 seedlings. **(A)** Cluster diagram of expression patterns of all differentially expressed genes (DEGs). The horizontal axis represents the clustering results for the R15, L28, and L30 materials, and the vertical axis represents the clustering of the DEGs. Each column represents one sample of the plant materials and each row represents a different gene. The color indicates the log (FPKM +1) expression level of the gene in the sample. **(B)** Venn diagram of DEGs. Each circle in the plot represents a differential grouping, and the intersection of the circles represents the common DEGs, with the corresponding number of genes identified in the set. **(C)** KEGG classification plot of DEGs. The vertical axis is the name of the KEGG metabolic pathway, and the horizontal axis is the number of genes annotated to the pathway as a proportion of the total number of genes annotated. The value beside each bar is the number of genes annotated for each pathway. **(D)** Expression patterns of 12 genes in R15, L28, and L30 determined by RNA-seq and qRT-PCR analyses. Error bars indicate the standard error (*n* = 3 biological replicates). *p < 0.05, **p < 0.01 (Student’s *t*-test).

To identify the regulatory pathway for AmCBF1-regulated plant dwarfing, we investigated the DEGs in R15, L28, and L30 in response to *AmCBF1* overexpression by RNA-seq. Cluster analysis of the DEGs demonstrated that the number of down-regulated genes affected by overexpression of *AmCBF1* was generally higher in L28 and L30 than in R15 (Figure 2A). A Venn diagram indicated that 780 DEGs were shared between R15 and L28, of which 309 were up-regulated and 471 were down-regulated in L28. There were 3,899 DEGs in common between R15 and L30, of which 1,424 were up-regulated and 2,475 were down-regulated in L30. Among these DEGs, 384 DEGs were common to L28 and L30 compared with R15 (Figure 2B). Analysis of Kyoto Encyclopedia of Genes and Genomes (KEGG) pathway enrichment revealed that the DEGs were mainly enriched in phytohormone signaling, DNA replication, starch and sucrose metabolism, carbon metabolism, and amino acid biosynthesis (Figure 2C). Subsequently, 12 DEGs from different metabolic pathways were randomly selected to verify the results of transcriptome sequencing, which suggested the RNA-seq results were reliable (Figure 2D). Notably, five *GhPP2C* genes were down-regulated in the transcriptome data (Supplementary Figure 1). These genes are important intermediate receptors in ABA signal transduction.

### Phylogenetic Analyses of Upland Cotton PP2C Family Members

**Figure 3:**
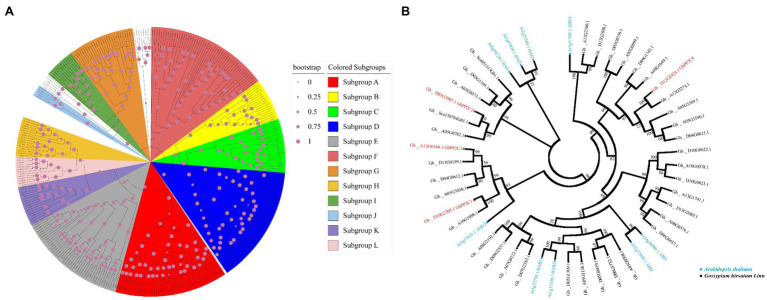
Phylogenetic relationships among PP2C family members. **(A)** Phylogenetic tree for PP2C family members of upland cotton and *Arabidopsis*. Sequences for PP2C family members of *Arabidopsis* were obtained from TAIR (http://www.arabidopsis.org). Subgroups A–L are as delimited in a previous study (Fuchs et al., 2013). Different subgroups are indicated by different colors. **(B)** Phylogenetic tree for subgroup A PP2C family members of upland cotton and *Arabidopsis*. Blue and black indicate *Arabidopsis* and upland cotton, respectively; red indicates the four *GhPP2C* genes detected in the transcriptome data. The evolutionary tree was constructed using the neighbor-joining method with MEGA7. The values at the nodes are bootstrap support percentages (*n* = 1,000 replicates).

Eighty PP2C family members have been identified in *Arabidopsis*, which are classified into 12 subgroups (Fuchs et al., 2013). To clarify the phylogenetic relationships of PP2C family members in upland cotton, the keyword “protein phosphatase 2C” was used as the query term in a search of the cottonFGD database. A total of 265 PP2C family members from the upland cotton genome were retrieved. Phylogenetic analysis showed that the upland cotton PP2Cs could be divided into 12 subgroups (A–L; Figure 3A). Among these subgroups, 39 PP2C family members were present in subgroup A and may be involved in ABA signaling. Interestingly, four GhPP2C proteins (GhPP2C1–GhPP2C4) belonged to subgroup A of the PP2C family. Among these proteins, GhPP2C1, GhPP2C2, and GhPP2C3 were most closely related to AtHAI1/2/3 and AtAHG3 in *Arabidopsis* (Figure 3B).

### Expression Analyses and Subcellular Localization of GhPP2Cs

**Figure 4:**
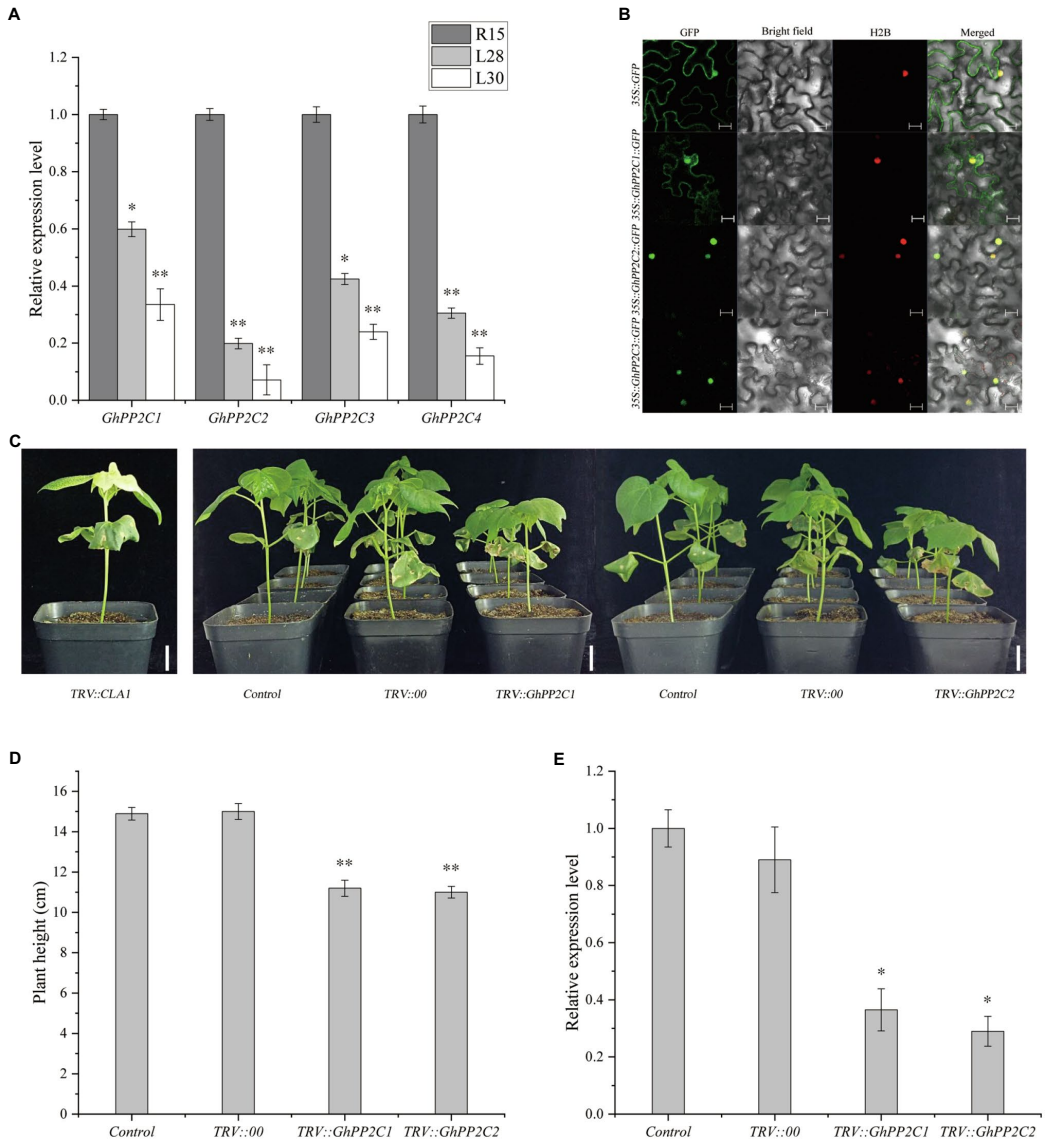
Preliminary analysis of *GhPP2C* gene functions. **(A)** Expression of four *GhPP2C* genes in cotton R15, L28, and L30 seedlings. **(B)** Subcellular localization of GhPP2C proteins. Fluorescence of GhPP2C1-GFP, GhPP2C2-GFP, and GhPP2C3-GFP fusion proteins and GFP (negative control) was detected at 488 nm by confocal microscopy with H2B-mCherry as the nucleolus marker; images were merged (Merged). Scale bar = 20 μm. **(C)** Plant phenotype after VIGS silencing of *GhPP2C1* or *GhPP2C2* in R15. TRV::*CLA1*, albino phenotype, positive control; Control, not treated; TRV::00, empty vector control. Scale bar = 2 cm. **(D)** Plant height after silencing of *GhPP2C1* and *GhPP2C2*. **(E)** Gene expression assay after silencing of G*hPP2C1* or *GhPP2C2*. Error bars indicate the standard error (*n* = 3 biological replicates). ^*^p < 0.05, ^**^p < 0.01 (Student’s *t*-test).

The expression levels of *GhPP2C1*–*GhPP2C4* were analyzed by qRT-PCR in three cotton materials (R15, L28, and L30). The expression levels were significantly lower in L28 compared with those in R15, and were extremely significantly decreased in L30. Compared with R15, the expression of these genes decreased by approximately 40–80% in L28 and 60–90% in L30 (Figure 4A). These results implied that the expression of these *GhPP2C* genes was negatively correlated with expression of AmCBF1.

To investigate the subcellular localization of the GhPP2C proteins, we first predicted the subcellular localization using the CELLO 2.5 online tool.^3^ The results predicted that GhPP2C1–GhPP2C3 were localized in the nucleus, whereas GhPP2C4 was localized in the chloroplast. Combined with the phylogenetic analysis, the subcellular localization of GhPP2C1–GhPP2C3 was further verified by fusion expression with GFP. The fluorescence signal of the GhPP2C1-GFP fusion protein was localized to the cell membrane and nucleus, whereas the GFP signal of the GhPP2C2-GFP and GhPP2C3-GFP fusion proteins was localized to the nucleus (Figure 4B). The 1302-GFP protein was used as a control.

### Silencing of *GhPP2C* Genes Reduces Plant Height

To further explore the function of *GhPP2C1*–*GhPP2C3* in regulating plant height, we separately silenced these genes in R15 using VIGS and observed whether the plant phenotype was altered. Silencing of *GhPP2C1* or *GhPP2C2* resulted in a significant decrease in growth rate, and the plant height was reduced by approximately 25% compared with that of the control (Figures 4C,D). The qRT-PCR analysis indicated that the relative expression level of these two genes was significantly decreased (by approximately 65 and 70%) in gene-silenced plants than in the control (Figure 4E). These results suggested that *GhPP2C* played a vital role in regulating cotton plant height. Silencing of *GhPP2C3* did not cause a significant change in phenotype, so no further studies were conducted on this gene (data not shown). TRV2::*CLA1* was used as a positive control (Figure 4C).

### AmCBF1 Binds Directly to the CRT/DRE Elements in the *GhPP2C* Promoter

**Figure 5:**
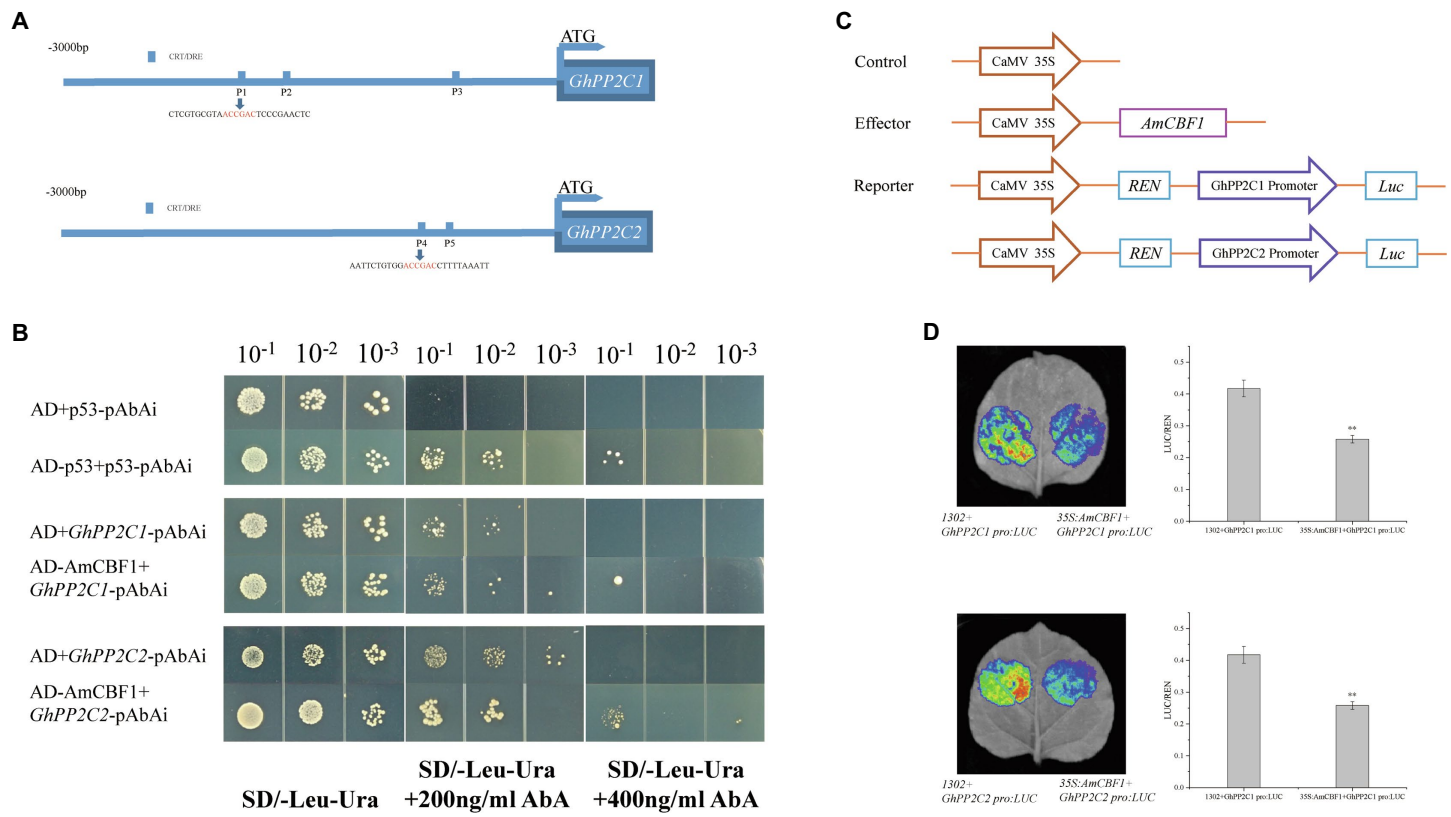
AmCBF1 transcription factor inhibition of the expression of *GhPP2C1* and *GhPP2C2*. **(A)** Schematic diagram of the CRT/DRE binding element in the 3,000 bp promoter upstream of the ATG start codon of *GhPP2C1* and *GhPP2C2*. **(B)** Yeast one-hybrid analysis showing binding of AmCBF1 to the promoter sequence of *GhPP2C1* or *GhPP2C2*. Yeast cells were cultured at serial dilutions of 1:10, 1:100, and 1:1,000 on SD/−Leu/−Ura medium supplemented with or lacking 400 ng ml^−1^ aureobasidin A. The pGADT7 vector was used as a negative control. **(C)** Schematic diagram of vector construction of the reporter and effector plasmids for transient transcriptional activity assays in tobacco. REN, *Renilla* luciferase; LUC, firefly luciferase. **(D)** Tobacco leaf live-imaging observations. Red indicates the strongest fluorescence signal intensity. Luciferase activity was normalized to the respective REN activity and expressed as relative expression. Error bars indicate the standard error (*n* = 3 biological replicates). ^**^p < 0.01 (Student’s *t*-test).

Overexpression of *AmCBF1* led to down-regulation in the expression level of *GhPP2C* genes, whereas silencing of the *GhPP2C* genes resulted in decreased plant height. These results suggested that there may be a regulatory relationship between AmCBF1 and GhPP2C proteins. The 3 kb promoter sequence upstream of *GhPP2C1* or *GhPP2C2* was further analyzed. Three or two CRT/DRE (A/GCCC/GAC) elements were identified and designated P1, P2, and P3 (upstream elements of *GhPP2C1*), and P4 and P5 (upstream elements of *GhPP2C2*; Figure 5A). A Y1H assay was conducted to verify whether AmCBF1 could bind to the promoter DNA fragments containing CRT/DRE elements. The yeast strains containing pGADT7 were unable to grow on SD/−Leu/−Ura medium supplemented with 400 ng ml^−1^ AbA, whereas the yeast strains harboring pGADT7-AmCBF1 were able to grow on this medium. Thus, AmCBF1 could directly bind to the CRT/DRE element in the promoter of *GhPP2C1* (containing P1, P2, and P3) or *GhPP2C2* (containing P4 and P5; Figure 5B). The binding properties between P1–P5 and AmCBF1 were also verified separately by means of a Y1H assay (Supplementary Figure 4A).

### AmCBF1 Represses Transcription of *GhPP2C1* or *GhPP2C2*

To verify how AmCBF1 regulates the transcription of *GhPP2C1* or *GhPP2C2*, a dual-luciferase assay was performed in tobacco. The relevant vectors were constructed (Figure 5C; Supplementary Figure 4B). When the suspension containing the reporter plasmid GhPP2C1 Pro:LUC, C1:LUC, C2:LUC, GhPP2C2 Pro:LUC or C3:LUC were, respectively, mixed with the suspension containing the effector plasmid 35S:AmCBF1 to inject tobacco, the LUC luminescence intensity was significantly reduced and the relative LUC/*Renilla* luciferase (REN) activity was significantly lower than that of the control (decreased by approximately 30–45%; Figure 5D; Supplementary Figure 4C). These results further suggested that AmCBF1 may act as a transcriptional repressor and inhibits the expression of *GhPP2C1* or *GhPP2C2*.

## Discussion

Plant architecture strongly influences the fiber quality and yield of cotton. New cotton varieties with a dwarf and compact growth phenotype not only perform well in terms of lodging resistance and photosynthetic efficiency, but also are suitable for mechanical harvesting to reduce labor costs (Bednarz et al., 2005; Dai et al., 2015). Overexpression of CBF/DREB transcription factors in a variety of plant species, including *Arabidopsis*, tobacco, tomato, and cotton, can lead to dwarfing (Zhang et al., 2004; Zhou et al., 2014, 2017; Ji et al., 2021). In the present study, L28 and L30 overexpressing *AmCBF1* both showed a dwarf phenotype in the greenhouse and in the field (Supplementary Figure 2). When *AmCBF1* expression in L28 and L30 was inhibited by VIGS, the plant height was partially recovered (Figures 1A,B). This result was consistent with previous research that silencing of *GhDREB1B* in the AS98 cotton mutant exhibited partial restoration to normal plant height (Ji et al., 2021). At the cellular level, the upper epidermal cells were observed by scanning electron microscopy before and after *AmCBF1* silencing. In addition to the change in size of epidermal cells, it was noteworthy that the epidermal cells were more tightly packed in L28 and L30 than in R15. The surface of the epidermal cells in L28 and L30 was less smooth than in R15, even after *AmCBF1* silencing, and this phenotype was not visibly restored (Figure 1D). We speculated that *AmCBF1* overexpression may affect the expression of genes associated with cell morphological structure, which leads to this change in cell morphology. Thus, the relationship between AmCBF1 and cell morphology requires further study.

To further investigate the genetic pathway of AmCBF1-regulated plant dwarfing, RNA-seq of R15, L28, and L30 seedlings was performed, and the genes differentially expressed in L28 or L30 in comparison with R15 were identified. Cluster analysis of the expression patterns of all DEGs revealed that most DEGs were significantly down-regulated in the overexpressed material, and more DEGs were down-regulated in L30 than in L28 (Figure 2A). A Venn diagram provided a visual representation of the number of DEGs common in the different materials (Figure 2B). However, the number of DEGs between R15 and L28 was 780, while 3,899 DEGs were found between R15 and L30. The possible reason for the huge differences in the number of DEGs may be due to the different insertion site of *AmCBF1*. A similar study revealed that in upland cotton overexpressing *DREB1B*, the number of up-regulated and down-regulated DEGs was approximately equal (Ji et al., 2021). We speculated that the difference in findings between these two studies was due to the different genes overexpressed in cotton or differences in the growth and development status of cotton seedlings used in transcriptome sequencing. The KEGG enrichment analysis of the DEGs revealed that most of the DEGs were enriched in phytohormone signaling pathways, and several genes were enriched in starch and sucrose metabolism, amino acid biosynthesis, carbon metabolism, and lignin biosynthesis pathways (Figure 2C). This result suggested that phytohormones may play an important role in plant dwarfing and was similar to the findings of previous reports (Chen et al., 2021; Sun et al., 2021).

Among the DEGs enriched in phytohormone signaling pathways, four *GhPP2C* genes were down-regulated. The function of *PP2C* genes has been widely studied in diverse plant species, such as *Arabidopsis*, rice, tomato, maize (*Zea mays*), and pepper (*Capsicum annuum*; Kerk et al., 2002; Singh et al., 2010; Kim et al., 2014; Wei and Pan, 2014; Liang et al., 2021). However, few studies have investigated the function of *PP2C* genes in cotton. In the present study, we analyzed the evolutionary relationships of PP2C family members in upland cotton and 265 putative *PP2C* genes were identified from the cottonFGD database. This result contrasted with a previous study in which 181 *PP2C* genes were identified in upland cotton (Shazadee et al., 2019). The difference might be due to the different cotton databases accessed or differences in retrieval methods. The 265 *GhPP2C* genes were classified into 12 subgroups of which 39 genes were classified in subgroup A. Interestingly, four of the five down-regulated *GhPP2C* genes identified by RNA-seq belonged to subgroup A. Among these genes, *GhPP2C1–GhPP2C3* were closely related to *AtHAI1/2/3* and *AtAHG3* (Figure 3B). Subcellular localization analysis revealed that GhPP2C1 was localized to the nucleus and cell membrane, whereas GhPP2C2 and GhPP2C3 were localized to the nucleus (Figure 4B). It has previously been reported that PP2C proteins are localized in the cytoplasm (Amir Hossain et al., 2018). The aforementioned results indicate that PP2C proteins may be localized in different subcellular locations to perform different functions. In the present study, silencing of *GhPP2C1* or *GhPP2C2* resulted in dwarfing (Figure 4C). Their functions in regulating plant height may be similar to those of *AtPP2CF1* (in *Arabidopsis*), *OsPP2C09* (in rice), and *SlPP2C3* (in tomato; Sugimoto et al., 2014).

Members of the CBF/DREB transcription factor subfamily interact with genes containing CRT/DRE elements in the promoter and either activate or repress gene expression (Magome et al., 2008; Tsutsui et al., 2009; Kuang et al., 2017; Zhou et al., 2017). The target genes of CBF/DREB transcription factors were significantly enriched in phytohormone signaling pathways, especially the ABA signaling pathway (Song et al., 2021). Whether *GhPP2C* genes are directly regulated by AmCBF1 and are involved in dwarfing in upland cotton has not been reported previously. In the present study, the upstream 3 kb promoter of *GhPP2C1* or *GhPP2C2* was analyzed. The Y1H assay verified that AmCBF1 was capable of binding to the CRT/DRE element in the promoter of *GhPP2C1* or *GhPP2C2* (Figure 5B; Supplementary Figure 4A). The dual-luciferase assay indicated that AmCBF1 directly inhibited transcription of *GhPP2C1* or *GhPP2C2* by binding to the CRT/DRE element in the promoter (Figure 5D; Supplementary Figures 4B,C). To our knowledge, the present study is the first to report that CBF-like transcription factors are involved in dwarfing of upland cotton by regulating the expression of *GhPP2C* genes.

There is increasing evidence that *PP2C* genes of subgroup A are crucial negative regulators of the ABA signaling pathway, and show distinct expression patterns and subcellular localization in plants (Saez et al., 2004; Nishimura et al., 2007; Umezawa et al., 2009; Zhang et al., 2012). Abscisic acid may exert various inhibitory effects in plant growth (Sharp and Lenoble, 2002; Chen et al., 2006; Zhao et al., 2015; Wang et al., 2018). The ABA–PYR/PYL/RCAR–PP2C complex is an important ternary complex that initiates signal transduction and activates the expression of downstream genes in the classical ABA signaling pathway (Hao et al., 2011; Antoni et al., 2012; Bhaskara et al., 2012; Chen et al., 2016). It is well known that GA and ABA have antagonistic effects in plants, jointly maintain the dynamic balance of plant hormones, and play roles in the growth and development of plants (Yoshida et al., 2014; Liu and Hou, 2018; Shu et al., 2018; Song et al., 2021; Tuan et al., 2021). Based on these findings and the present results, we hypothesized that AmCBF1 overexpression may affect ABA signaling by down-regulating the expression of *GhPP2C1* or *GhPP2C2*, thereby leading to cotton dwarfing by disrupting the balance of ABA and GA.

## Conclusion

**Figure 6:**
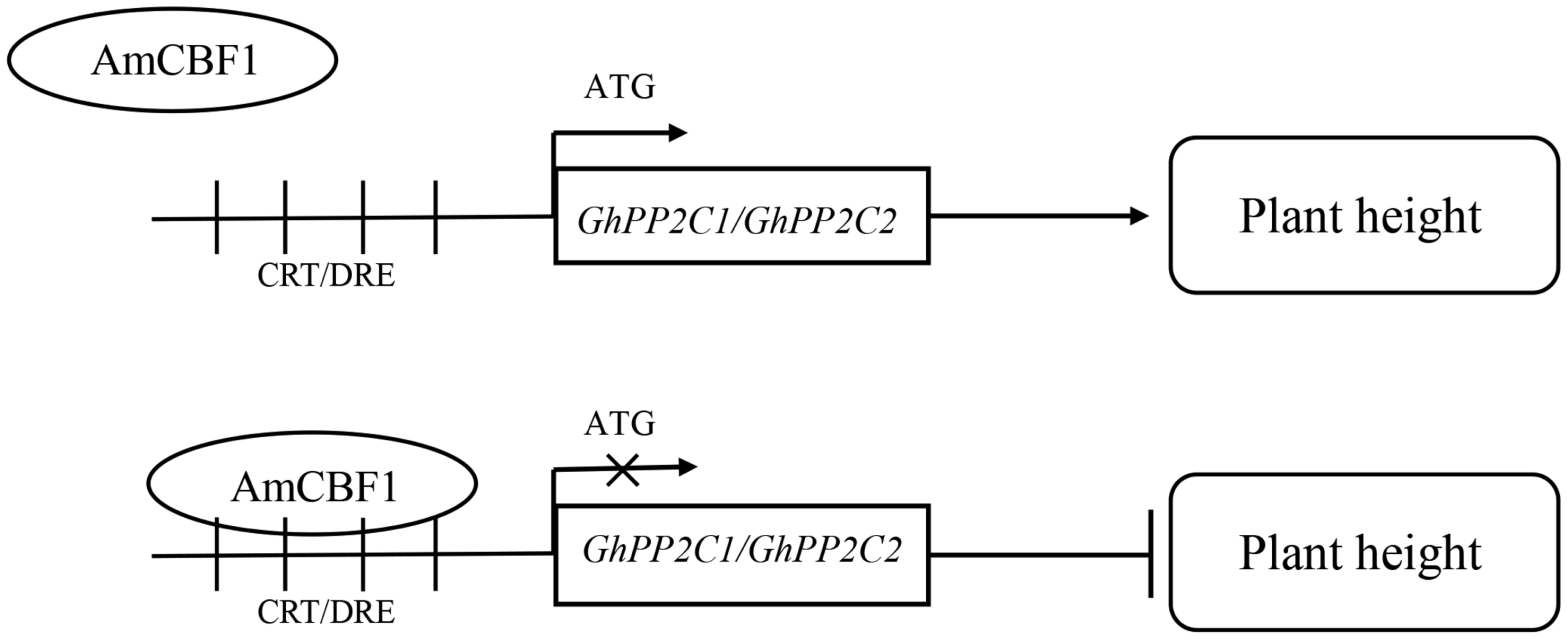
Schematic diagram of a working model for AmCBF1 regulation of *GhPP2C* genes. The transcription factor AmCBF1 inhibits expression of *GhPP2C* genes and negatively regulates the growth of upland cotton seedlings by binding to CRT/DRE elements. Bar, inhibition.

Overexpression of the AmCBF1 transcription factor in upland cotton affected plant growth. *GhPP2C1* and *GhPP2C2* were identified among the DEGs detected by RNA-seq, and silencing of these two genes caused a dwarf phenotype in upland cotton. We verified that AmCBF1 could bind to CRT/DRE elements and negatively regulate the expression of *GhPP2C* genes, thereby regulating plant height. Based on these findings, we propose a molecular model for coordinated regulation of plant height by AmCBF1 and *GhPP2C* genes (Figure 6). The present findings expand our knowledge of the regulatory mechanism of dwarfing in cotton, and might contribute to the molecular breeding of cotton cultivars with a dwarf phenotype.

## Data Availability Statement

The datasets presented in this study can be found in online repositories. The transcriptome sequencing data can be found in the NCBI database under accession no. PRJNA791756.

## Author Contributions

HG designed the experiments. JL and LW performed the experiments. JL and LW performed the data analysis. QZ, CM, XS, and HC provided valuable suggestions on the research design and improvement of the manuscript. JL and HG wrote the manuscript. All authors read and approved the final manuscript.

## Funding

This work was supported by Central Public-interest Scientific Institution Basal Research Fund (nos. Y2022PT22 and Y2022ZK25).

## Conflict of Interest

The authors declare that the research was conducted in the absence of any commercial or financial relationships that could be construed as a potential conflict of interest.

## Publisher’s Note

All claims expressed in this article are solely those of the authors and do not necessarily represent those of their affiliated organizations, or those of the publisher, the editors and the reviewers. Any product that may be evaluated in this article, or claim that may be made by its manufacturer, is not guaranteed or endorsed by the publisher.

## Footnotes

^1^ https://cottonfgd.org/

^2^ https://www.arabidopsis.org/

^3^ http://cello.life.nctu.edu.tw/
