# Supplementary material for: AmCBF1 Transcription Factor Regulates Plant Architecture by Repressing GhPP2C1 or GhPP2C2 in Gossypium hirsutum
Source: Front Plant Sci. 2022 May 30;13:914206. doi: 10.3389/fpls.2022.914206 (PMC9197424; doi:10.3389/fpls.2022.914206)
Supplement: Supplementary file 1 [file Data_Sheet_1.docx]

## Supplementary Tables

**Supplementary Table 1**. Primers used in this study

| **Primer name** | **Primer sequences (5'->3')** | **Description** |
| --- | --- | --- |
| AmCBF1 RT F | GTGCTGACGTGGAATTTGATGA | qPCR |
| AmCBF1 RT R | ATTTCACGGGTTGGGGTTTCT | qPCR |
| Q1 RT F | TGGCATTCCTTTCATCTGCGGT | qPCR |
| Q1 RT R | CGCGAACCGAAATGAGCTTTCC | qPCR |
| Q2 RT F | TGCTTAGGATCAACTGCGACCG | qPCR |
| Q2 RT R | ACAGACTTTATCAAGCGGGGCG | qPCR |
| Q3 RT F | GGATGGCTGTGGCCTTGAGAAT | qPCR |
| Q3 RT R | ACAGCCCTGCTGAAGGTAGAGT | qPCR |
| Q4 RT F | CCATTGATGATGACTGCTTTGC | qPCR |
| Q4 RT R | ATTGCTCGAGTCAGAGCTTGTA | qPCR |
| Q5 RT F | TGTGGCCATTGCATGTGTTAGC | qPCR |
| Q5 RT R | TTTCCACCCCCATTTACGCCAG | qPCR |
| Q6 RT F | GTGGAGCATGTCCTTCGTCTGG | qPCR |
| Q6 RT R | GCAGGAGTGGGAATAAGTCCGC | qPCR |
| Q7 RT F | TCTCCACCACCAAGCCACTACT | qPCR |
| Q7 RT R | CAATCAGGTTTGGACGGTCGGA | qPCR |
| Q8 RT F | GGGTCACTGCTACAACGATTTGC | qPCR |
| Q8 RT R | ACAGCTGGTCTCTGTGTTTCCA | qPCR |
| Q9 RT F | CACCACGAATCGACAGTCCGAG | qPCR |
| Q9 RT R | ACATCCGTCCACATGGTCATCG | qPCR |
| Q10 RT F | ACGGACTTCTTGAATCCGAACGA | qPCR |
| Q10 RT R | TCGTTTTTGGTACACCGAGCGT | qPCR |
| Q11 RT F | CCGATGAGCAAGTGTTTGTGCC | qPCR |
| Q11 RT R | GTGCTCGACCATCTGAAAGCCT | qPCR |
| Q12 RT F | TGATCTCGATCTCGTCCTCCCG | qPCR |
| Q12 RT R | TCGTCGACCACAAACGGAAGTC | qPCR |
| GhPP2C1 RT F | GAAGTTCGGAATGGCTTCCGGT | qPCR |
| GhPP2C1 RT R | CTTCCACTCCACCGCCTTCAAA | qPCR |
| GhPP2C2 RT F | CCGGAAACGCAAGAACCTG | qPCR |
| GhPP2C2 RT R | GGATGTAACTCTGAATCGATTTCAGG | qPCR |
| GhPP2C3 RT F | AACAAGGTTAACGGAGATCAGG | qPCR |
| GhPP2C3 RT R | AAGTCAAACCAAACTTCGGCAA | qPCR |
| GhPP2C4 RT F | CTCGCCAGAGAAGGTTGAAGT | qPCR |
| GhPP2C4 RT R | TACCAATAACCGACACCACCC | qPCR |
| GhUBQ RT F | AGCTCGGATACGATTGATAACG | qPCR |
| GhUBQ RT R | GAAGACGAAGAACAAGGGGAAG | qPCR |
| 1302-F | ACTATCCTTCGCAAGACCCTTC | Subcellular localization |
| 1302-R | TGTGCCCATTAACATCACCATC | Subcellular localization |
| 1302-GhPP2C1-F | acgggggactcttgaccatggACATGAATAAGAAGTTCGTGGAAGATG | Subcellular localization |
| 1302-GhPP2C1-R | tactagtcagatctaccatggAGAGCAATTCATCGGGTCG | Subcellular localization |
| 1302-GhPP2C2-F | acgggggactcttgaccatggacATGGCGGAGATCTGTTACGG | Subcellular localization |
| 1302-GhPP2C2-R | tactagtcagatctaccatggACGTGGCTCTCCTTAGATCCAC | Subcellular localization |
| 1302-GhPP2C3-F | acgggggactcttgaccatggACATGGCTGGAGTTTGCTGTG | Subcellular localization |
| 1302-GhPP2C3-R | tactagtcagatctaccatggATAACTGTTGATTTTCCTTCAAATCAACAACG | Subcellular localization |
| pTRV2-F | TACTTAGGAAGTGGCTTGAC | VIGS assay |
| pTRV2-R | CCGATCTCAAACAGTCTATAC | VIGS assay |
| VIGS-AmCBF1 -F | aaggttaccgaattctctagaCAAGAAGACCAGGATTTGGCTC | VIGS assay |
| VIGS-AmCBF1 -R | gagacgcgtgagctcggtaccAAGTAATGTGTTGGCGACATC | VIGS assay |
| VIGS-GhPP2C1 F | aaggttaccgaattctctagaTGGAAGATGAAAAGGAACGGC | VIGS assay |
| VIGS-GhPP2C1 R | gagacgcgtgagctcggtaccCCTTGGCGTTTACAGTCCATTC | VIGS assay |
| VIGS-GhPP2C2 F | aaggttaccgaattctctagaCTCCATCTGAAGCTGACAACG | VIGS assay |
| VIGS-GhPP2C2 R | gagacgcgtgagctcggtaccAACGCTCTCTGCACCTCATC | VIGS assay |
| VIGS-GhPP2C3 F | aaggttaccgaattctctagaATCTCGATCTCGTCCTCCCG | VIGS assay |
| VIGS-GhPP2C3 R | gagacgcgtgagctcggtaccGCTTCTCTCCATGGTTCGCT | VIGS assay |
| 1302-AmCBF1-F | CGGGGGACTCTTGACCATGTTTTCCTTCAATCATTTTTCCGATCCAC | Dual-luciferase assay |
| 1302-AmCBF1-R | TAGTCAGATCTACCATAATTGAATAACTCCACAATGAAACTTCAGCA | Dual-luciferase assay |
| Luc-F | tcgaggtcgacggtatcgataag | Dual-luciferase assay |
| Luc-R | ttccatcttccagcggatagaatg | Dual-luciferase assay |
| Luc-GhPP2C1-F | ggtggagatcgaattccatggCGAGAAGGTCCTTAACATTTGAGC | Dual-luciferase assay |
| Luc-C1-F | ggtggagatcgaattccatggGCCAAATTAGCTTTTGGAGAAAAGG | Dual-luciferase assay |
| Luc-C2-F | ggtggagatcgaattccatggCTAGTTAAGCCAATTTAATTCGCGCC | Dual-luciferase assay |
| Luc-GhPP2C1-R | tgtttttggcgtcttccatggTAATTTTACCGTTCCATTAGAAAATAGACCTCG | Dual-luciferase assay |
| Luc-GhPP2C2-F | ggtggagatcgaattccatggATCTTGAGGTAAGAGTTCGGTGG | Dual-luciferase assay |
| Luc-C3-F | ggtggagatcgaattccatggATGGACATGGGGACCTACAAG | Dual-luciferase assay |
| Luc-GhPP2C2-R | tgtttttggcgtcttccatggAACAAGAACTAGAACCACACCATTATTATCAT | Dual-luciferase assay |
| pGADT7-F | AATACGACTCACTATAGGGCGAG | Y1H assay |
| pGADT7-R | GGCCAAGATTGAAACTTAGAGGAG | Y1H assay |
| pAbAi-F | TGCTCCTTCCTTCGTTCTTCC | Y1H assay |
| pAbAi-R | CCATCTCGAAAAAGGGTTTGCC | Y1H assay |
| AD-AmCBF1-F | gccatggaggccagtgaattcATGTTTTCCTTCAATCATTTTTCCGATCCAC | Y1H assay |
| AD-AmCBF1-R | cagctcgagctcgatggatccTTAAATTGAATAACTCCACAATGAAACTTCAGCA | Y1H assay |
| pAbAi-GhPP2C1-F | cttgaattcgagctcggtaccCGAGAAGGTCCTTAACATTTGAGC | Y1H assay |
| pAbAi-GhPP2C1-R | agcacatgcctcgaggtcgacTAATTTTACCGTTCCATTAGAAAATAGACCTTCG | Y1H assay |
| pAbAi- P1-F | CCTCGTGCGTAACCGACTCCCGAACTCG | Y1H assay |
| pAbAi- P1-R | TCGACGAGTTCGGGAGTCGGTTACGCACGAGGGTAC | Y1H assay |
| pAbAi- P2-F | CGAAAAGGAAAACCCACAACTAATTCAG | Y1H assay |
| pAbAi- P2-R | TCGACTGAATTAGTTGTGGGTTTTCCTTTTCGGTAC | Y1H assay |
| pAbAi- P3-F | CTCACTGATTGGCCCACATGTCTACATG | Y1H assay |
| pAbAi- P3-R | TCGACATGTAGACATGTGGGCCAATCAGTGAGGTAC | Y1H assay |
| pAbAi-GhPP2C2-F | cttgaattcgagctcggtaccTCCTCTTCCTTTTGGGGGCCA | Y1H assay |
| pAbAi-GhPP2C2-R | agcacatgcctcgaggtcgacAACAAGAACTAGAACCACACCATTATTATCAT | Y1H assay |
| pAbAi- P4-F | CTAAATTCTGTGGACCGACCTTTTAAATTAAG | Y1H assay |
| pAbAi- P4-R | TCGACTTAATTTAAAAGGTCGGTCCACAGAATTTAGGTAC | Y1H assay |
| pAbAi- P5-F | CGGGTTTGATTGGCCCACGACATCCTAAGCGG | Y1H assay |
| pAbAi -P5-R | TCGACCGCTTAGGATGTCGTGGGCCAATCAAACCCGGTAC | Y1H assay |

**Supplementary Table 2**. List of genes randomly detected by qPCR

| Gene id | Gene description | R15 vs L28 | R15 vs L30 |
| --- | --- | --- | --- |
| (Q1) Gh_D13G0129 | Cytokinin dehydrogenase 6 | 3.489222547 | 1.441552057 |
| (Q2) Gh_A01G1605 | alcohol dehydrogenase 1 | 1.822478155 | 1.199756501 |
| (Q3) Gh_D06G0690 | Inositol oxygenase 4 | -1.929450509 | 1.820041472 |
| (Q4) Gh_D12G1387 | Valine--tRNA ligase, chloroplastic/mitochondrial 2 | 1.654016237 | 1.503199703 |
| (Q5) Gh_A10G1143 | Probable acyl-activating enzyme 16, chloroplastic | 1.0290298 | 1.218100688 |
| (Q6) Gh_D05G1218 | Palmitoyl-acyl carrier protein thioesterase, chloroplastic | 1.123702071 | 2.042208987 |
| (Q7) Gh_D08G1164 | Hyoscyamine 6-dioxygenase | -4.993181874 | -2.118658569 |
| (Q8) Gh_A07G0261 | WRKY transcription factor 18 | -2.429474096 | -2.852694449 |
| (Q9) Gh_A06G1585 | Probable WRKY transcription factor 70 | -3.066271915 | -1.941208335 |
| (Q10) Gh_A08G1993 | Alcohol dehydrogenase-like 6 | -2.07831591 | -1.609281838 |
| (Q11) Gh_A08G0863 | Plastidial pyruvate kinase 4, chloroplastic | -2.450769125 | -1.404589914 |
| (Q12) Gh_D10G2305 | Protein phosphatase 2C 37 | -1.428761593 | -1.07381982 |
