# Supplementary figures and images for: AmCBF1 Transcription Factor Regulates Plant Architecture by Repressing GhPP2C1 or GhPP2C2 in Gossypium hirsutum
Source: Front Plant Sci. 2022 May 30;13:914206. doi: 10.3389/fpls.2022.914206 (PMC9197424; doi:10.3389/fpls.2022.914206)

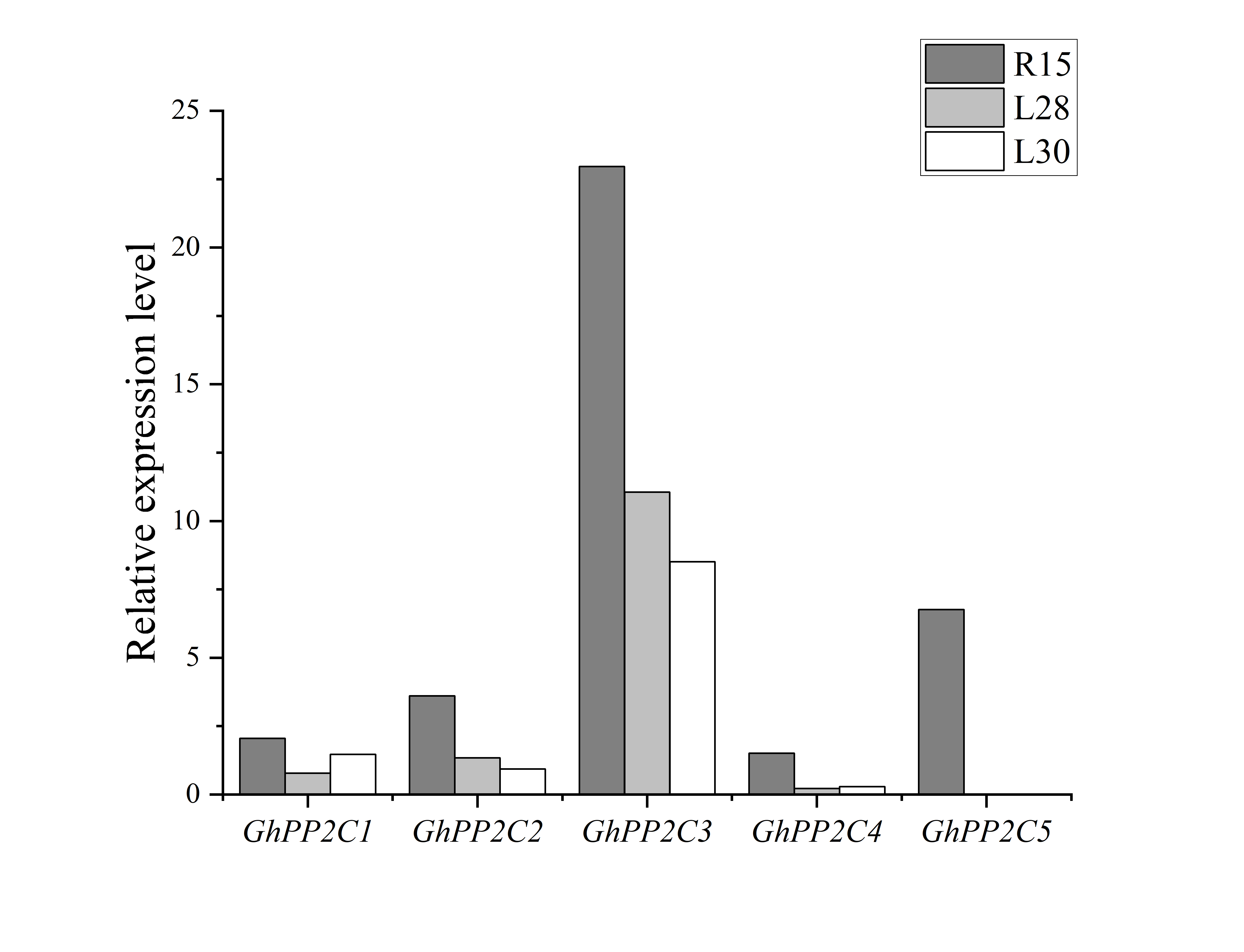

Supplement: Supplementary file 2 [file Image_1.TIF]

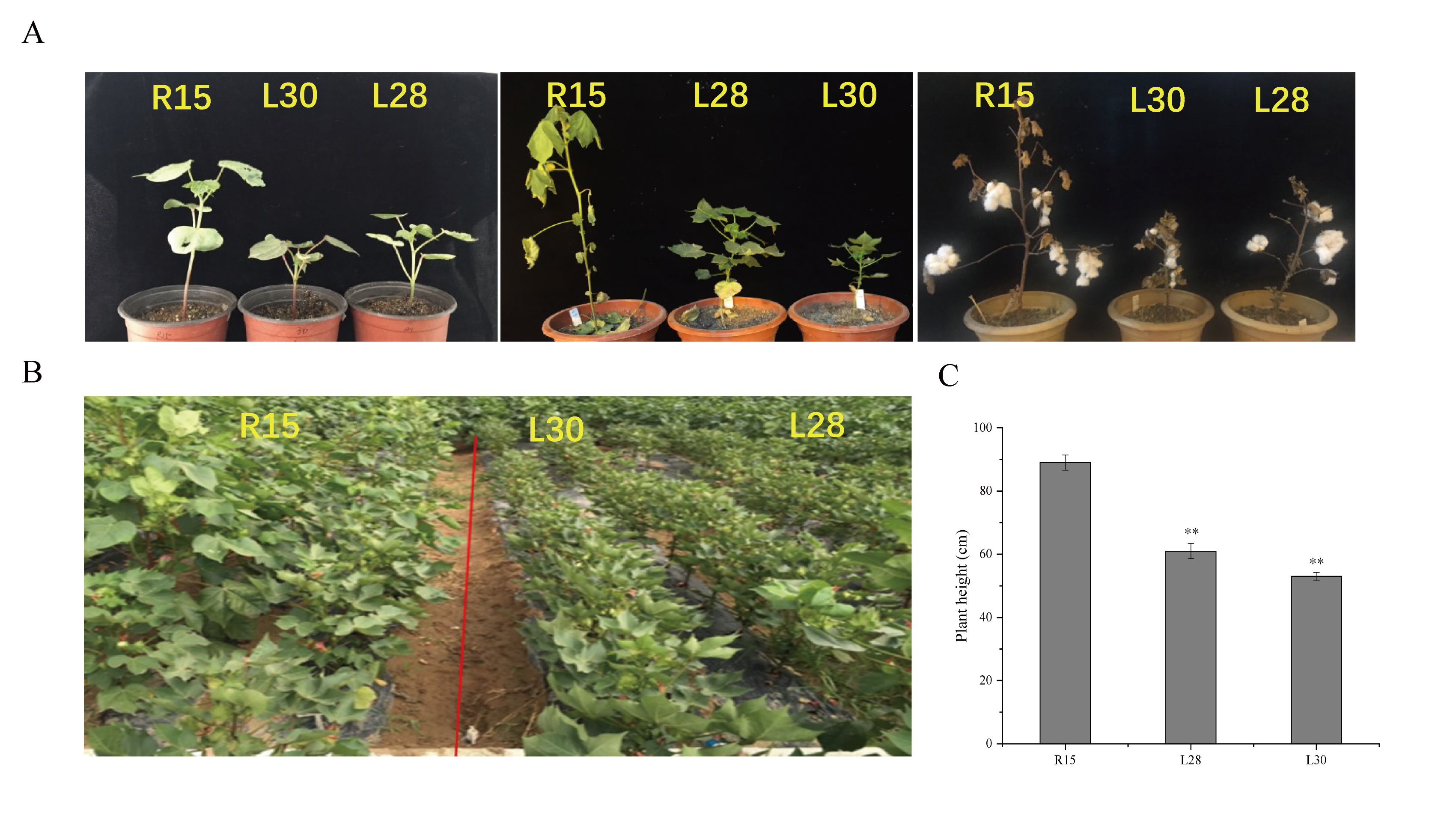

Supplement: Supplementary file 3 [file Image_2.TIF]

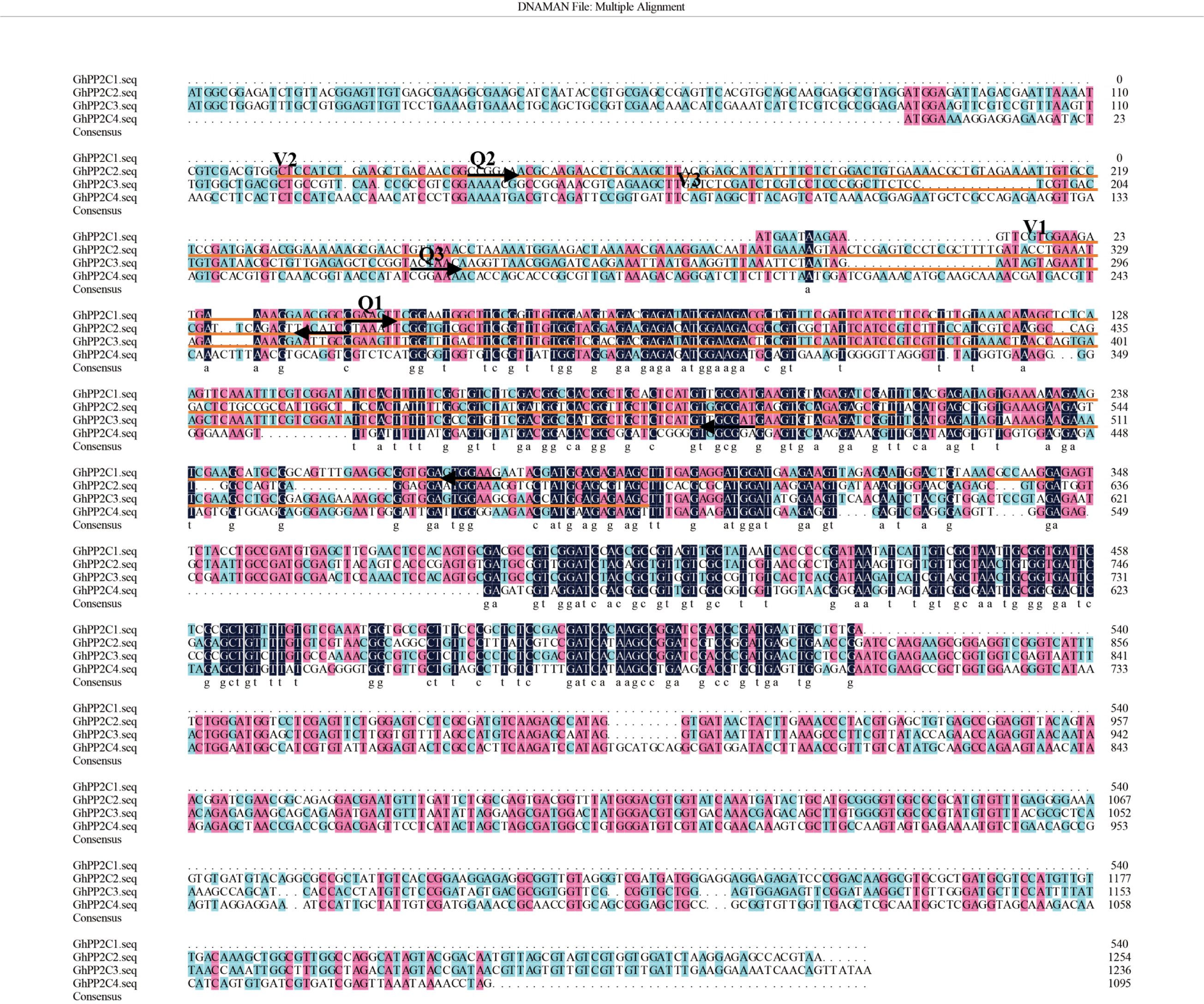

Supplement: Supplementary file 4 [file Image_3.TIF]

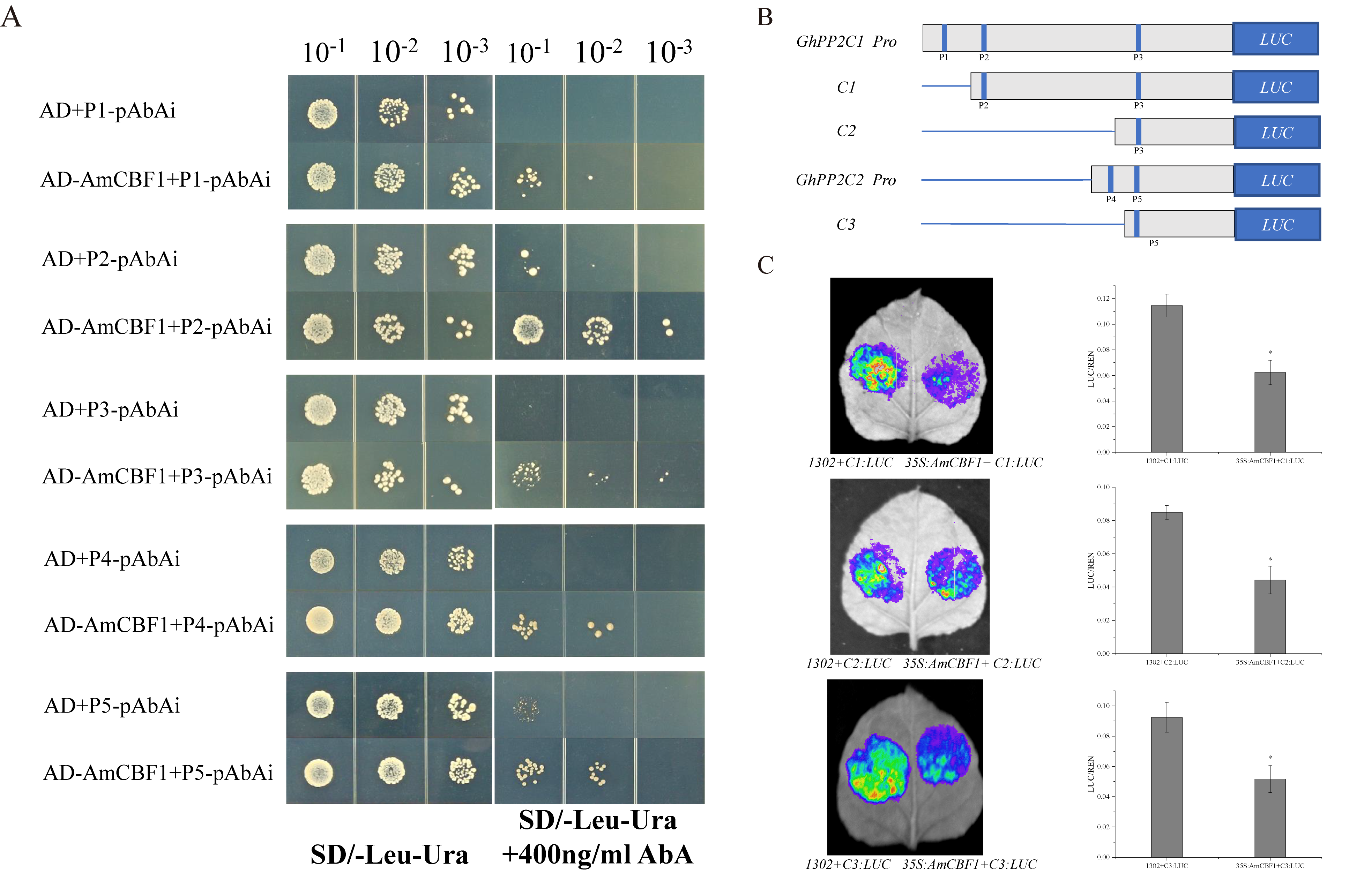

Supplement: Supplementary file 5 [file Image_4.TIF]
